# Prevalence of Constipation in Elderly and Its Association With Dementia and Mild Cognitive Impairment: A Cross-Sectional Study

**DOI:** 10.3389/fnins.2021.821654

**Published:** 2022-01-24

**Authors:** Fei Wang, Min Fei, Wen-Zheng Hu, Xiao-Dan Wang, Shuai Liu, Yan Zeng, Jin-Hong Zhang, Yang Lv, Jian-ping Niu, Xin-ling Meng, Pan Cai, Yang Li, Bao-zhi Gang, Yong You, Yan Lv, Yong Ji

**Affiliations:** ^1^Clinical College of Neurology, Neurosurgery and Neurorehabilitation, Tianjin Medical University, Tianjin, China; ^2^Department of Neurology, Yuncheng Central Hospital, Shanxi Medical University, Yuncheng, China; ^3^Department of Neurology, Beijing Tiantan Hospital, Capital Medical University, China National Clinical Research Center for Neurological Diseases, Beijing, China; ^4^Tianjin Key Laboratory of Cerebrovascular and Neurodegenerative Diseases, Department of Neurology, Tianjin Dementia Institute, Tianjin Huanhu Hospital, Tianjin, China; ^5^Brain Science and Advanced Technology Institute, Wuhan University of Science and Technology, Wuhan, China; ^6^Department of Neurology, Cangzhou People’s Hospital, Cangzhou, China; ^7^Department of Geriatrics, The First Affiliated Hospital of Chongqing Medical University, Chongqing, China; ^8^Department of Neurology, The Second Affiliated Hospital of Xiamen Medical College, Xiamen, China; ^9^Department of Neurology, Affiliated Traditional Chinese Medicine Hospital of Xinjiang Medical University, Urumqi, China; ^10^Dementia Clinic, Affiliated Hospital of Zunyi Medical University, Zunyi, China; ^11^Department of Neurology, The First Hospital of Shanxi Medical University, Taiyuan, China; ^12^Department of Neurology, The First Affiliated Hospital of Harbin Medical University, Harbin, China; ^13^Department of Neurology, The Second Affiliated Hospital of Hainan Medical University, Haikou, China; ^14^Department of Neurology, Hainan General Hospital, Haikou, China

**Keywords:** constipation, dementia, mild cognitive impairment, prevalence, Chinese elderly

## Abstract

**Background:**

Constipation and dementia have similar epidemiological characteristics. Changes in intestinal flora and characteristics of the brain-gut axis play roles in the pathogeneses of the two diseases, suggesting that there may be a close connection between the two. Most of the studies on constipation in dementia patients have focused on the population with α-synucleinopathies [Parkinson’s disease dementia (PDD), dementia with Lewy bodies (DLB)]. Few studies have reported the prevalence of constipation in all-cause dementia and mild cognitive impairment (MCI) populations.

**Objective:**

To assess the prevalence of constipation in patients with all-cause dementia and MCI subtypes and to explore the association between constipation with dementia and MCI subtypes.

**Methods:**

From May 2019 to December 2019, we conducted a population-based cross-sectional survey. A total of 11,743 participants aged 65 or older from nine cities in China were surveyed. Participants underwent a series of clinical examinations and neuropsychological measurements. Constipation, dementia, MCI and MCI subtype were diagnosed according to established criteria through standard diagnostic procedures.

**Results:**

The overall age- and sex-adjusted prevalence of constipation in individuals aged 65 years and older was 14.8% (95% CI, 14.6–15.0). The prevalence rates of constipation were19.2% (95% CI, 17.3–21.0), 19.1% (95% CI, 16.8–21.5), 14.4% (95% CI, 12.8–15.9), and 13.8% (95% CI, 13.0–14.6) in the dementia, non-amnestic (na)-MCI, amnestic (a)-MCI and normal cognition populations, respectively. Multivariate logistic regression analysis showed that higher prevalence of constipation was associated with dementia (*p* = 0.0.032, OR = 1.18, 95% CI: 1.02–1.38) and na-MCI (*p* = 0.003, OR = 1.30, 95% CI: 1.09–1.54).

**Conclusion:**

The present study found a high prevalence of constipation in elderly individuals in China, and higher in patients with dementia and na-MCI.

## Introduction

Constipation is common in among elderly individuals and may significantly affect quality of life. It mainly manifests as persistent hard or lumpy stools, reduced frequency of defecation (less than three times per week) or a sensation of incomplete evacuation, and digital stimulation is often required to relieve impacted stool (Bernard et al., 2009; Drossman and Hasler, 2016). Based on a meta-analysis conducted in 2011, the global pooled prevalence of constipation in the community-dwelling adult population was 14% (Bytzer et al., 2001). It is generally accepted that the prevalence of constipation increases with age (Vazquez Roque and Bouras, 2015), and can reach 33.5% in community-dwelling people over 60 years old (Bharucha et al., 2013). A study in the Chinese population showed that the total prevalence of constipation was 8.2%, and a higher rate was reported in people over 60 years old (18.1–35.8%) (Chu et al., 2014).

As an important manifestation of autonomic nerve dysfunction, constipation is closely related to dementia (Idiaquez and Roman, 2011; Allan, 2019; Mendoza-Velásquez et al., 2019). Constipation has been observed in a variety of neurodegenerative diseases, especially dementia with Lewy bodies (DLB) and Parkinson’s disease (PD), in which constipation is the most frequently reported early symptom (Savica et al., 2018). Moreover, a previous study found that PD patients with constipation were more likely to progress to Parkinson’s disease dementia (PDD) than those without constipation (De Pablo-Fernandez et al., 2017). However, the occurrence of constipation is limited in patients with Alzheimer’s disease (AD) and frontotemporal lobar degeneration (FTLD) (Idiaquez and Roman, 2011; Jensen-Dahm et al., 2015; Kim et al., 2019; Fu et al., 2020). At present, the pathogeneses of constipation and dementia are unclear, but they both share similar risk factors, such as age, sex, economic status, dietary structure, etc. (Ferri et al., 2005; Ziegler-Graham et al., 2008; Castellani et al., 2010; Zhang et al., 2018), and many studies have confirmed that brain-gut axis dysfunction and changes in intestinal flora can affect the occurrence of the two diseases (Barbara et al., 2005; Rhee et al., 2009; Attaluri et al., 2010; Carabotti et al., 2015; Cattaneo et al., 2017). Therefore, we hypothesized that constipation and dementia are closely related or even mutually causal.

The epidemiological characteristics of constipation in general communities have been reported in a large number of studies, but there have not been any large-sample studies in people with cognitive impairment, particularly those with the early stages of dementia, including mild cognitive impairment (MCI). Therefore, the purpose of this survey was to understand the prevalence of constipation in patients with dementia and MCI subtypes and to explore the association between constipation and cognitive dysfunction.

## Materials and Methods

### Study Design and Participants

**FIGURE 1:**
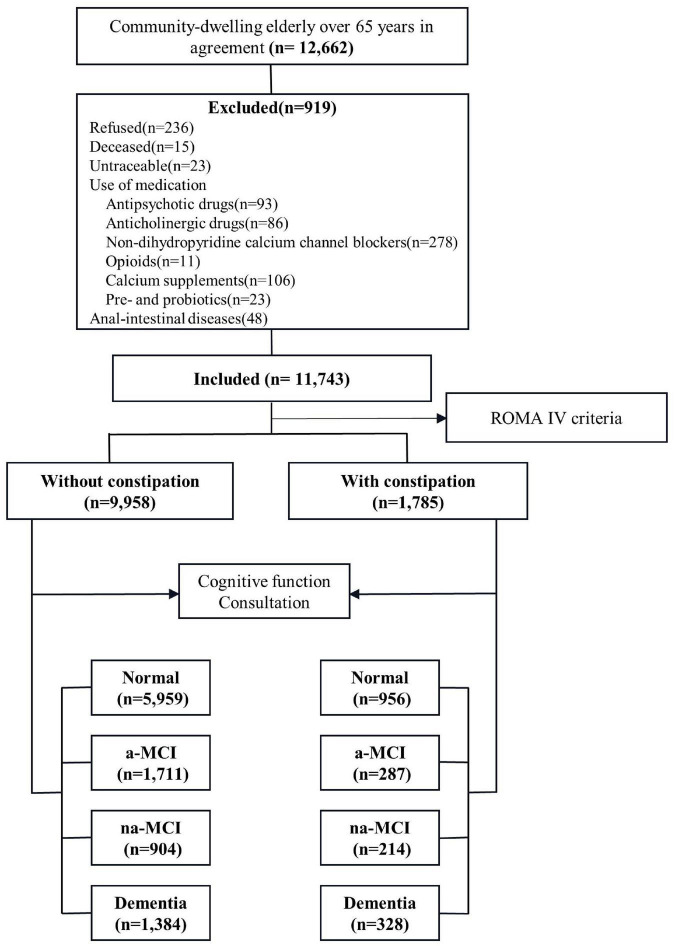
Flow of participants.

Our study involved a cross-sectional survey of the general population from May 2019 to December 2019. Data were collected using a multistage stratified cluster sampling design (Figure 1). According to 2010 census data, we considered geographical region, economic development level and population size and selected nine cities as centers (Tianjin, Cangzhou, Harbin, Taiyuan, Urumqi, Chongqing, Xiamen, Zunyi and Haikou). In the second stage, we selected a total of 10 urban areas and 11 counties for which the per capita gross domestic products (GDPs) were closest to the median GDPs of urban and rural populations. Finally, a total of 29 communities and 45 townships (villages) were selected from the selected urban areas and counties, and the permanent resident populations of the selected communities and townships ranged from 1,000 to 2,000.

The participant selection criteria were selected as follows: (1) at least 1 year of registered residence in the target area before the start date of the survey, (2) Han nationality and aged 65 years or older, (3) care provided by a caregiver with complete cognitive function and an ability to consent to the survey and complete the questionnaire. The exclusion criteria were as follows: refusal to participate, inability to be tracked, prevalence of a life-threatening disease, death or prevalence of a disease such as hearing or vision deficiency, using products and medications affecting bowel movements (antipsychotic drugs, anticholinergic drugs, non-dihydropyridine calcium channel antagonists, opioids, calcium supplements, pre- and probiotics), anal-intestinal diseases that may cause constipation (trauma, tumor, abdominal or pelvic surgery).

The study protocol was approved by the Huanhu Hospital (Tianjin, PRC) Research Ethics Committee (2019-40). Written informed consent was provided by each participant or responsible caregiver before participation in the study.

### Assessment and Diagnosis

#### Training

Study investigators were primarily junior neurologists and senior neurology graduate students. The expert panel consisted of senior neurologists and neuropsychologists with expertise in cognitive impairment. All interviewers and experts received the same training on neuropsychological assessment and diagnosis and were retrained every 2 months. The interrater reliability for cognitive tests and diagnostic procedures was set at > 0.90, and this was retained throughout the study.

#### Diagnostic Procedures

First, we conducted individual, semi-structured interviews with participants and their caregivers at their residence. All interviews were recorded and lasted for approximately 2 h. Detailed information on sociodemographic characteristics, lifestyle and medical history was collected. Data on lifestyle habits, including smoking, alcohol consumption, social activity, and marital status, as well as medical history, including hypertension, diabetes mellitus, heart disease, stroke, and headache history, were gathered.

Second, we conducted defecation and neurological examinations using standardized protocols. Constipation was assessed using a questionnaire based on the Bristol Stool Scale and Rome Criteria VI (Drossman and Hasler, 2016). Cognition was assessed using the Mini-Mental State Examination (MMSE), and social functioning was assessed using the activities of daily living (ADL) assessment.

When the MMSE scores were less than or equal to the cutoff (17 for illiterate individuals, 20 for those with 1–6 years of education, 24 for individuals with ≥ 7 years education), a third screening survey as well as physical and neurological examinations were conducted, medical records were reviewed, and clinical Dementia Score Questionnaire (CDR) scores were calculated. All data were collected by trained staff in accordance with standard procedures.

All information was reviewed by an expert panel and interviewers, and diagnoses were made at the end of each working day. When consensus was not reached, an expert returned to the residence to re-examine and re-evaluate the participant for a final diagnosis.

#### Diagnostic Criteria

Constipation was determined on the basis of the Rome IV criteria. Participants’ cognitive function status was classified as normal, MCI, or dementia. Subjects with normal cognition scored 0 points on the global CDR. Dementia was diagnosed according to the Diagnostic and Statistical Manual of Mental Disorders, 5th edition. Participants with evidence of impaired cognition but not dementia were classified as having MCI according to the clinical criteria recommended by the National Institute on Aging and Alzheimer’s Association (NIA-AA) workgroup (Albert et al., 2011). MCI can be classified into two main clinical subtypes, amnestic MCI (aMCI) or non-amnestic MCI (na-MCI), based on whether memory is impaired.

### Statistical Analysis

Data were analyzed using the Statistical Program for Social Sciences, version 23.0 (IBM Corporation, Armonk, NY, United States). Demographics are presented as numbers and frequency distributions for categorical variables as appropriate. Estimates of the prevalence of constipation in a-MCI, na-MCI and dementia populations were calculated separately for the overall population. Wilcoxon’s rank sum test, Kruskal–Wallis test and the chi-square test were used to determine significant differences between individuals with and without constipation. The association between constipation and cognitive function was investigated by using multivariable logistic regression models and models that were adjusted for age, sex, education level, and other variables and to evaluate potential confounding effects among risk factor variables. A p-value less than 0.05 was considered statistically significant.

## Results

### Characteristics of the Sampled Populations

A total of 11,743 residents participated in the survey, including 1,785 with constipation and 9,958 without constipation. The median age was 73 years old, and there were 6,576 women (56.0%) and 7,272 rural people (61.9%) (Supplementary Table 1). We observed demographic differences between the two groups. The median age of the constipation group was older (age was 74). The constipation group had a higher proportion of women living in rural areas with hypertension, diabetes, stroke, headache and heart disease. However, there was no significant difference in marital status, solitude, smoking or drinking between the two groups. There were significant differences in MMSE score, CDR score and ADL score between the two groups, and the performance of the constipation group was worse (Table 1).

**TABLE 1:** Characteristics of the study population by constipation.

Characteristics	Without constipation (*n* = 9,958)	With constipation (*n* = 1,785)	χ^2^/Z	*P*-value
Age, (years), median (IQR)	72.0 (68.0, 78.0)	74.0 (70.0, 80.0)^b^	77.11	<0.001
Sex, female, *n* (%)	5,521 (55.4)	1,055 (59.1)^b^	8.23	0.004
Marital status, *n* (%)				
Single	95 (1.0)	19 (1.1)	1.43	0.232
Married	7,532 (75.6)	1,322 (74.1)		
Divorced/widowed	2,331 (23.4)	444 (24.9)		
Residence location, *n* (%)				
Urban	3,904 (39.2)	567 (31.8)^b^	35.54	<0.001
Rural	6,054 (60.8)	1,218 (68.2)^b^		
Education level, (years), median (IQR)	6.0 (3.0, 9.0)	6.0 (2.0, 9.0)^b^	25.03	<0.001
Illiterate	2,011 (20.2)	402 (22.5)	34.67	<0.001
Primary school	4,461 (44.8)	886 (49.6)^b^		
Middle school and above	3,486 (35.0)	497 (28.7)^b^		
Live alone, *n* (%)	1,155 (11.6)	207 (11.6)	0.00	0.998
Smoking, *n* (%) *	2,502 (25.1)	446 (25.0)	0.02	0.900
Alcohol consumption, *n* (%) **	2,234 (22.4)	398 (22.3)	0.02	0.898
Hypertension, *n* (%)	4,312 (43.3)	934 (52.3)^b^	49.86	<0.001
Diabetes mellitus, *n* (%)	1,328 (13.3)	372 (20.8)^b^	68.85	<0.001
Stroke/TIA, *n* (%)	1,142 (11.5)	315 (17.6)^b^	53.18	<0.001
Cerebral hemorrhage, *n* (%)	160 (1.6)	41 (2.3)^a^	4.29	0.038
Headache, *n* (%)	430 (4.3)	177 (9.9)^b^	96.76	<0.001
Heart disease, *n* (%)	1,412 (14.2)	443 (24.8)^b^	128.79	<0.001
MMSE score, median (IQR)	26.0 (22.0, 28.0)	25.0 (20.0, 28.0)^b^	7.79	<0.001
CDR score, median (IQR)	0.0 (0.0, 0.5)	0.0 (0.0, 0.5)^b^	−5.69	<0.001
ADL score, median (IQR)	20.0 (20.0, 21.0)	20.0 (20.0, 23.0)^b^	−8.13	<0.001

*Results are shown as n (%) for the chi-square tests and as the median (IQR) for Wilcoxon’s rank sum test. TIA, transient ischemic attack; MMSE, Mini-Mental State Examination; CDR, clinical dementia rating; ADL, activities of daily living; IQR: Inter quartile range.*

*Significance was indicated by the following p values: ^a^P < 0.05 vs. Without constipation; ^b^P < 0.01 vs. Without constipation.*

*Smoking was defined as having smoked at least 400 cigarettes.

**Alcohol consumption was defined as drinking at least 0.1 drink per day for 1 year or more, with one drink equal to 10 g pure alcohol.

### Prevalence of Constipation in Different Cognitive Statuses

**FIGURE 2:**
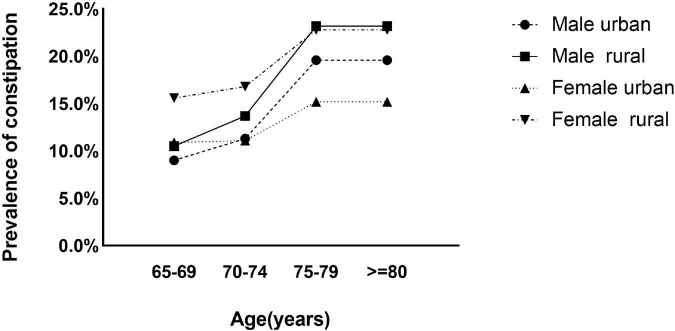
Prevalence of constipation by age and sex categories in urban and rural populations.

The crude overall prevalence of constipation was 15.2% (95% CI, 14.6–15.8), the age- and sex-adjusted prevalence rate was estimated to be 14.8% (95% CI, 14.6–15.0) in individuals aged 65 years and older. The prevalence rates in women and rural populations were significantly higher than those in men (16.0 vs. 14.1%) and urban populations (16.7 vs. 12.7%), respectively (Supplementary Table 2). In the age, sex and residence location subgroups, the prevalence of constipation increased with age in people aged 65 to 79 years (Figure 2).

**FIGURE 3:**
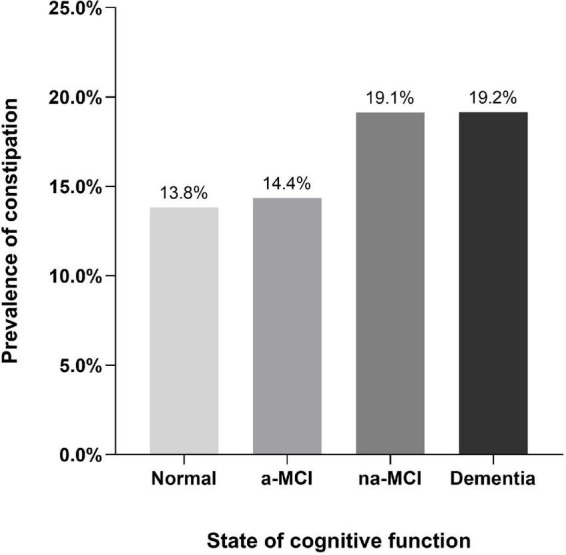
Prevalence of constipation by state of cognitive function.

According to cognitive function status, the dementia and na-MCI groups had higher prevalence rates of constipation (19.2%, 95% CI, 17.3–21.0 and 19.1%, 95% CI, 16.8–21.5, respectively), and there was no statistically significant difference between the groups. There was no significant difference in the prevalence of constipation between the a-MCI group and the normal cognitive function group (13.8%, 95% CI, 13.0–14.6 and 14.4%, 95% CI, 12.8–15.9, respectively) (Supplementary Table 2), but the prevalence rates in the two groups were significantly lower those in the dementia and na-MCI groups (Figure 3).

### Association Between Constipation and Cognitive Function

The results of the multivariate regression analyses are presented in Table 2. In the unadjusted model, constipation it showed that higher prevalence of constipation was associated with dementia and na-MCI. After adjustment for confounders, this positive association was retained in models 1, 2, and 3 (Table 2).

**TABLE 2:** OR and 95% CI for the presence of constipation in subjects of different cognitive function groups by multivariate logistic regression model.

	Normal	a-MCI		na-MCI		Dementia	
		OR (95% CI)	*P-value*	OR (95% CI)	*P*-value	OR (95% CI)	*P*-value
Crude model	ref.	1.05 (0.91–1.21)	0.540	1.48 (1.25–1.74)	**<0.001**	1.48 (1.29–1.70)	**<0.001**
Model 1	ref.	1.00 (0.87–1.16)	0.991	1.47 (1.25–1.74)	**<0.001**	1.28 (1.11–1.48)	**0.001**
Model 2	ref.	1.03 (0.89–1.19)	0.715	1.41 (1.20–1.67)	**<0.001**	1.21 (1.04–1.40)	**0.012**
Model 3	ref.	1.07 (0.92–1.24)	0.408	1.30 (1.09–1.54)	**0.003**	1.18 (1.02–1.38)	**0.032**

*Model 1, was adjusted for age, sex. Model 2, was adjusted for age, sex, marital status, residence location, education level and live alone. Model 3, was adjusted for age, sex, marital status, residence location, education level, live alone, smoking, alcohol consumption, hypertension, stroke/TIA, cerebral hemorrhage, headache, and heart disease. OR, odds ratio. CI, Confidence interval. a-MCI, amnestic mild cognitive impairment. na-MCI, non-amnestic mild cognitive impairment. Bold values indicate statistical significance.*

## Discussion

Constipation is a global disease that affects quality of life. Our study found that the prevalence of constipation in mainland Chinese people aged over 65 years old was 14.8%, and it was higher in women, which is consistent with previous epidemiological studies and systematic reviews (Markland et al., 2013; Andy et al., 2016). In this study, the prevalence of constipation tended to increase with age, although the association is still controversial. A meta-analysis published in 2021 showed that there was no significant correlation between constipation and age (Werth and Christopher, 2021), but research in the Chinese population has shown such a correlation (Chu et al., 2014). Our survey found that the prevalence of constipation in rural residents was significantly higher than that in urban residents, and similar results have been shown in other studies. Additionally, the education level of people with constipation was significantly lower than that of people without constipation. Our results showed the proportion of people with a junior high school education or above was lower in people without constipation than in those with constipation, and the proportion of a low education level (primary school and below) among people with constipation was significantly higher than that among people without constipation. In addition, the proportions of vascular risk factors (hypertension, diabetes, ischemic stroke, cerebral hemorrhage, headache and heart disease) in the constipation group were significantly higher than those in the non-constipation group. The above epidemiological characteristics of constipation have been confirmed in many studies (Sorouri et al., 2010; Suares and Ford, 2011; Chu et al., 2014; Ebling et al., 2014; Moezi et al., 2018), and these characteristics are similar to those associated with dementia (Jia et al., 2020), that was also confirmed based on our study (Supplementary Table 3). After analyzing the epidemiological characteristics of the two diseases, some studies have proposed that there may be a correlation between constipation and dementia (Zhang et al., 2018).

The specific pathogeneses of constipation and dementia are still unclear, but an increasing number of studies has shown that there is an important connection between them (Idiaquez and Roman, 2011; Chen et al., 2020; Leta et al., 2021). The gut is connected to the brain through the gut-brain axis (Rhee et al., 2009); that is, the central nervous system communicates with the enteric nervous system, intestinal mucosa and muscle layer through two-sided (afferent and efferent) pathways. Pathological changes in any component of the gut-brain axis may affect the activity of the intestine and lead to constipation. At the same time, pathological studies have demonstrated that α-synuclein deposits can originate from the intestinal plexus and develop along the vagus nerve, eventually reaching the brain, resulting in the development of dementia (Mendoza-Velásquez et al., 2019).

In recent years, research on the role of intestinal microbiota in constipation and dementia has increased. The intestinal microbiota can affect gastrointestinal motility by affecting intestinal myometrial electrical activity, affecting the release of bacterial fermentation end-products and intestinal neuroendocrine factors, resulting in constipation (Barbara et al., 2005). A previous study found that the intestinal flora of adult patients with constipation varied significantly; the abundances of *Bifidobacterium* and *Lactobacillus* were decreased significantly (Khalif et al., 2005), the abundances of *Prevotella* and *Firmicutes* were increased (Zhu et al., 2014), and those of *Coprococcus*, *Roseburia* and *Faecalibacterium* were also increased in patients with constipation (Pryde et al., 2002; Sokol et al., 2008). Similarly, intestinal microbiota can affect the occurrence of dementia by directly inducing intestinal endocrine cells to synthesize a variety of neurotransmitters, neuropeptides, hormones and immune modulators; acting on the brain through the brain-gut axis; and regulating advanced neural functions, including cognition (Welcome, 2018). When the intestinal flora changes, it can cause the production of toxic bacterial metabolites in the intestine to increase, resulting in increased intestinal permeability, increased blood-brain barrier permeability, nervous system inflammation and neurodegenerative changes (Sawada et al., 2003; Epstein and Chapman, 2008; Alkasir et al., 2017; Erdő et al., 2017; Friedland and Chapman, 2017). Large abundance reductions in *Lactobacillus* and *Bifidobacterium* and increases *infecal bacilli*, *Faecalibacterium* and *Roseburia* have been found in the intestinal flora of dementia patients as well as constipation patients (Welcome, 2018).

The above studies indicate that the damage of the brain-gut axis and the changes of the flora are important pathophysiological basis for the occurrence of constipation and dementia, and there may be the same or interaction mechanism. This study confirmed the correlation between constipation and dementia. Compared with that in the population with normal cognitive function, the prevalence of constipation in the population with all-cause dementia was significantly higher (19.2 vs. 13.8%), and the higher rates of constipation was associated with dementia after controlling for age, sex, residence, education level and related vascular risk factors.

There is still no effective treatment for dementia, and the pathological process occurs many years before obvious cognitive impairment. MCI is considered to be the state preceding dementia. The research results showed that not all MCI patients progressed to dementia. Due to the existence of cognitive reserve, a considerable number of MCI patients will not develop dementia (Nelson et al., 2021). Therefore, research on MCI is of great significance, but there are few studies on constipation in patients with MCI and its subtypes. The results of this study showed that the prevalence of constipation in the na-MCI population was significantly higher than those in the cognitively normal population and the a-MCI population, and there was no significant difference in the prevalence of constipation between the a-MCI and normal populations. After controlling for possible interactive factors, the multivariate logistic regression model showed that constipation was significantly associated with na-MCI and dementia compared with normal cognitive function, but there was no significant correlation between constipation and a-MCI.

a-MCI has long been associated with progression to AD, and its pathological mechanism is the deposition of amyloid protein and nerve fiber tangle caused by tau protein (Hughes et al., 2011; Knopman et al., 2015). Although biological research has suggested that intestinal flora can promote the occurrence of AD by directly inducing amyloid and neuro-inflammatory responses, there are still few studies focused on AD and constipation, and clinical research results suggest that the progression of AD will not be significantly accompanied by changes in the prevalence of constipation. Therefore, we speculate that the intestinal flora may affect the pathological process of AD through the brain-gut axis during the a-MCI stage, but has no significant effect on constipation, even when the course of the disease progresses to the AD stage many years later. DLB, PDD, and other α-synuclein diseases together account for a considerable proportion of dementia cases (Boström et al., 2007), and in a large proportion of patients, na-MCI will progress to DLB and PDD, which are early-stage synucleinopathathies. The deposition of α-synuclein can start in the intestinal nerve, resulting in early symptoms of constipation (Beach et al., 2010). Therefore, it is reasonable that the prevalence of constipation in the na-MCI population was higher than that in the a-MCI population. As the deposition of α-synuclein gradually progressed to the brain, severe cognitive dysfunction gradually appeared, which evolved from MCI to dementia. At dementia stage, the symptoms of constipation may enter a plateau. Therefore, patients with na-MCI will almost have the same rates of constipation as with dementia. Our research also confirmed this situation, the prevalence of constipation in the na-MCI group was significantly higher than that of the normal population and a-MCI population, and there was no statistical difference in the prevalence of constipation compared with the dementia population.

The results of multiple logistic regression analysis also showed corresponding associations. Therefore, we believe that constipation in MCI stage may clinically assist in the identification of the MCI subtype.

The advantage of this study is that it is the latest large-scale, multicenter and national epidemiological study on constipation in those over the age of 65 in China. In addition, we conducted an in-depth analysis of the relationship between constipation and cognitive impairment and provide new evidence for the association between constipation and cognitive impairment. We also conducted sub analyses by MCI subtype and found differences in the association between constipation and different subtypes of MCI. Inevitably, this study has several limitations. First, because our study was a retrospective study, we do not know the exact ages at constipation, dementia onset and the changes in symptoms overtime, so it is impossible to determine the causal relationship between constipation and dementia and na-MCI. Second, in our study, we have not further distinguished the types of dementia and assessed the etiology of MCI in patients. It is impossible to more accurately explain the relationship between constipation and more detailed types of dementia/MCI. Third, more factors that can affect constipation and cognitive dysfunction need to be evaluated and analyzed. Therefore, a prospective cohort study with more influencing factors and detailed classification of dementia and MCI is necessary.

In conclusion, according to the above research results, we found that constipation is a common chronic disease in elderly individuals aged over 65 years, higher prevalence of constipation was associated with dementia and na-MCI.

## Data Availability Statement

The raw data supporting the conclusion of this article will be made available by the authors, without undue reservation.

## Ethics Statement

The studies involving human participants were reviewed and approved by Huanhu Hospital (Tianjin, PRC) Research Ethics Committee. The patients/participants provided their written informed consent to participate in this study.

## Author Contributions

FW: writing the initial draft, design of methodology, investigation, and revising the manuscript. MF: analyzing data, investigation, and revising the manuscript. W-ZH: visualization and interpretation of data, Investigation, and revising the manuscript. X-DW, YZ, J-HZ, YGL, J-PN, X-LM, PC, YLi, B-ZG, YY, and YL: investigation, data management of the center, and revising the manuscript. SL: investigation, project administration, and revising the manuscript. YJ: design of study, funding acquisition, supervision, and revising the manuscript. All authors contributed to the article and approved the submitted version.

## Conflict of Interest

The authors declare that the research was conducted in the absence of any commercial or financial relationships that could be construed as a potential conflict of interest.

## Publisher’s Note

All claims expressed in this article are solely those of the authors and do not necessarily represent those of their affiliated organizations, or those of the publisher, the editors and the reviewers. Any product that may be evaluated in this article, or claim that may be made by its manufacturer, is not guaranteed or endorsed by the publisher.
