# Supplementary material for: Prevalence of Constipation in Elderly and Its Association With Dementia and Mild Cognitive Impairment: A Cross-Sectional Study
Source: Front Neurosci. 2022 Jan 24;15:821654. doi: 10.3389/fnins.2021.821654 (PMC8819140; doi:10.3389/fnins.2021.821654)
Supplement: Supplementary file 1 [file Table_1.DOCX]

Supplementary Material

# Defecation Questionnaire

1. Whether there is recurrent abdominal pain before and after defecation?(Yes/No)
2. Time of symptom onset( years)
3. Frequency of occurrence in the past 3 months( %)
4. Frequency of occurrence in the past 6 months( %)
5. Frequency of occurrence in the past 12 months or more ( %)
6. Weather you have increased bowel movements when abdominal pain occurs?
7. Time of symptom onset( years)
8. Frequency of occurrence in the past 3 months( %)
9. Frequency of occurrence in the past 6 months( %)
10. Frequency of occurrence in the past 12 months or more ( %)
11. Weather you have increased bowel movements when abdominal pain occurs?
12. Time of symptom onset( years)
13. Frequency of occurrence in the past 3 months( %)
14. Frequency of occurrence in the past 6 months( %)
15. Frequency of occurrence in the past 12 months or more ( %)
16. Whether there are the following symptoms occur in defecation?
17. Straining (Yes/No)
18. Time of symptom onset( years)
19. Frequency of occurrence in the past 3 months( %)
20. Frequency of occurrence in the past 6 months( %)
21. Frequency of occurrence in the past 12 months or more ( %)
22. Sensation of incomplete evacuation (Yes/No)
23. Time of symptom onset( years)
24. Frequency of occurrence in the past 3 months( %)
25. Frequency of occurrence in the past 6 months( %)
26. Frequency of occurrence in the past 12 months or more ( %)
27. Sensation of anorectal obstruction/blockage (Yes/No)
28. Time of symptom onset( years)
29. Frequency of occurrence in the past 3 months( %)
30. Frequency of occurrence in the past 6 months( %)
31. Frequency of occurrence in the past 12 months or more ( %)
32. Manual maneuvers to facilitate (Yes/No)
33. Time of symptom onset( years)
34. Frequency of occurrence in the past 3 months( %)
35. Frequency of occurrence in the past 6 months( %)
36. Frequency of occurrence in the past 12 months or more ( %)
37. The number of spontaneous bowel movements per week
38. In the past 3 months( )
39. In the past 6 months( )
40. In the past 12 months or more( )
41. Distribution of the fecal forms according to **Bristol Stool Scale**
42. In the past 3 months: Type 1( %), Type 2( %), Type 3( %), Type 4( %), Type 5( %), Type 6( %) Type 7( %)
43. In the past 6 months: Type 1( %), Type 2( %), Type 3( %), Type 4( %), Type 5( %), Type 6( %) Type 7( %)
44. In the past 12 months or more: Type 1( %), Type 2( %), Type 3( %), Type 4( %), Type 5( %), Type 6( %) Type( %)
45. Frequency of loose stools in defecation without using laxatives
46. In the past 3 months( %)
47. In the past 6 months( %)
48. In the past 12 months or more( %)

# Supplementary Tables

**Table 1** Characteristics of the study population (n=11,743)

| **Variable** | **Median ( IQR) or n (%)** |
| --- | --- |
| Age , (years) | 73(69~78) |
| Sex |  |
| Male | 5167(44.0) |
| Female | 6576(56.0) |
| Marital status |  |
| Single | 114(1.0) |
| Married | 8854(75.4) |
| Divorced/widowed | 2775(23.6) |
| Residence location |  |
| Urban | 4471(38.1) |
| Rural | 7272(61.9) |
| Education level , (years) | 6(3~9) |
| Illiterate | 2413(20.5) |
| Primary school | 5347(45.5) |
| Middle school and above | 3983(33.9) |
| Live alone |  |
| Yes | 1362(11.6) |
| No | 10381(88.4) |
| Smoking* |  |
| Yes | 2948(25.1) |
| No | 8795(74.9) |
| Alcohol consumption** |  |
| Yes | 2632(22.4) |
| No | 9111(77.6) |
| Hypertension |  |
| Yes | 5246(44.7) |
| No | 6497(55.3) |
| Diabetes mellitus |  |
| Yes | 1700(14.5) |
| No | 10043(85.5) |
| Stroke/TIA |  |
| Yes | 1457(12.4) |
| No | 10286(87.6) |
| Cerebral hemorrhage |  |
| Yes | 201(1.7) |
| No | 11542(98.3) |
| Headache |  |
| Yes | 607(5.2) |
| No | 11136(94.8) |
| Heart disease |  |
| Yes | 1855(15.8) |
| No | 9888(84.2) |
| MMSE score | 26(21~28) |
| ADL score | 20(20~22) |
| CDR score | 0(0~0.5) |

TIA, transient ischemic attack; MMSE, Mini-Mental State Examination; CDR, clinical dementia rating; ADL ,activities of daily living; IQR: Inter quartile range. *Smoking was defined as having smoked at least 400 cigarettes. **Alcohol consumption was defined as drinking at least 0.1 drink per day for 1 year or more, with one drink equal to 10 g pure alcohol.

**Table 2** Prevalence of constipation (n=11,743)

|  | **% (95% CI)** | **χ^2^** | **P** |
| --- | --- | --- | --- |
| Overall |  |  |  |
| Crude | 15.2(14.6~15.8) |  |  |
| Standardized^*^ | 14.8(14.6~15.0) |  |  |
| Age (years) |  |  |  |
| 65-69 | 12.3(11.2~13.4)^b,c^ | 85.5 | **<0.001** |
| 70-74 | 14.0(12.9-15.2)^e^ |  |  |
| 75-79 | 15.7(14.2-17.3)^f^ |  |  |
| >=80 | 20.7(19.1-22.3) |  |  |
| Sex |  |  |  |
| Male | 14.1(13.2-15.1) | 8.23 | **0.004** |
| Female | 16.0(15.2-16.9) |  |  |
| Residence location |  |  |  |
| Urban | 12.7(11.7-13.7) | 35.54 | **<0.001** |
| Rural | 16.7(15.9-17.6) |  |  |
| Cognitive function |  |  |  |
| normal | 13.8(13.0-14.6)^g,h^ | 45.51 | **<0.001** |
| a-MCI | 14.4(12.8-15.9)^i,j^ |  |  |
| na-MCI | 19.1(16.8-21.5) |  |  |
| dementia | 19.2(17.3-21.0) |  |  |

Results are shown as n (%) for the chi-square tests.Statistical significance in different age groups(P< 0.05):a, 65-69 vs. 70-74; b, 65-69 vs. 75-79; c, 65-69 vs. >=80; d, 70-74 vs. 75-79; e, 70-74 vs. >=80; f, 75-79 vs. >=80. Statistical significance in different cognitive status(P< 0.05): g, normal vs. na-MCI; h, normal vs. dementia; i, a-MCI vs. na-MCI; j, a-MCI vs. Dementia. *Adjusted, for age and sex location. CI: Confidence interval. a-MCI: amnestic mild cognitive impairment. na-MCI: non-amnestic mild cognitive impairment. Bold values indicate statistical significance.

**Table 3** Characteristics of the study population by cognitive function (n=11,743)

| **Characteristics** | **Normal**  **n= 6915** | **a-MCI**  **n= 1998** | **na-MCI**  **n=1118** | **Dementia**  **n=1712** | **χ^2^/Z** | **P** |
| --- | --- | --- | --- | --- | --- | --- |
| Age, (years), median(IQR) | 72(68~76)^a,b,c^ | 73(69~79)^d,e^ | 73(69~78)^f^ | 77(71.0~84) | 455.9 | <0.001 |
| Sex, n (%) |  |  |  |  |  |  |
| Male | 3137(45.4)^b,c^ | 910(45.5)^d,e^ | 582(52.1)^f^ | 538(31.4) | 146.5 | <0.001 |
| Female | 3778(54.6)^b,c^ | 1088(54.5)^d,e^ | 536(47.9)^f^ | 1174(68.6) |  |  |
| Marital status, n (%) |  |  |  |  |  |  |
| Single | 48(0.7)^d^ | 21(1.1) | 13(1.2) | 32(1.9) | 225.4 | <0.001 |
| Married | 5470(79.1)^a,d^ | 1476(73.9)^e^ | 848(75.8)^f^ | 1060(61.9) |  |  |
| Divorced/widowed | 1397(20.2)^a,d^ | 501(25.1)^e^ | 257(23.0)^f^ | 620(36.2) |  |  |
| Residence location, n (%) |  |  |  |  |  |  |
| Urban | 2846(41.2)^a,b,c^ | 918(45.9)^d,e^ | 303(27.1) | 404(23.6) | 289.6 | <0.001 |
| Rural | 4069(58.8)^a,b,c^ | 1080(54.1)^d,e^ | 815(72.9) | 1308(76.4) |  |  |
| Education level , (years), median(IQR) | 6(5~9)^a,b,c^ | 6(4~9)^d,e^ | 6(4~9)^f^ | 3(0~6) | 596.4 | <0.001 |
| Illiterate | 1294(18.7)^a,b,c^ | 274(13.7)^e^ | 152(13.6)^f^ | 693(40.5) | 611.3 | <0.001 |
| Primary school | 3036(43.9)^a,b^ | 985(49.3)^d,e^ | 631(56.4)^f^ | 695(40.6) |  |  |
| Middle school and above | 2585(37.4)^b,c^ | 739(37.0)^d,e^ | 335(30)^f^ | 324(18.9) |  |  |
| Live alone, n (%) |  |  |  |  |  |  |
| Yes | 742(10.7)^a,c^ | 266(13.3) | 123(11.0) | 231(13.5) | 17.2 | 0.001 |
| No | 6173(89.3)^a,c^ | 1732(86.7) | 995(89.0) | 1481(86.5) |  |  |
| Smoking^*^, n (%) |  |  |  |  |  |  |
| Yes | 1763(25.5)^b,c^ | 493(24.7)^d,e^ | 384(34.3)^f^ | 308(18.0) | 97.6 | <0.001 |
| No | 5152(74.5)^b,c^ | 1505(75.3)^d,e^ | 734(65.7)^f^ | 1404(82.0) |  |  |
| Alcohol consumption^**^, n (%) |  |  |  |  |  |  |
| Yes | 1618(23.4)^b,c^ | 419(21.0)^d,e^ | 341(30.5)^f^ | 254(14.8) | 104.8 | <0.001 |
| No | 5297(76.6)^b,c^ | 1579(79.0)^d,e^ | 777(69.5)^f^ | 1458(85.2) |  |  |
| Hypertension, n (%) |  |  |  |  |  |  |
| Yes | 3216(46.5)^a,b,c^ | 847(42.4) | 455(40.7) | 728(42.5) | 24.0 | <0.001 |
| No | 3699(53.5)^a,b,c^ | 1151(57.6) | 663(59.3) | 984(57.5) |  |  |
| Diabetes mellitus, n (%) |  |  |  |  |  |  |
| Yes | 970(14.0) | 315(15.8) | 158(14.1) | 257(15.0) | 4.3 | 0.230 |
| No | 5945(86.0) | 1683(84.2) | 960(85.9) | 1455(85.0) |  |  |
| Stroke/TIA, n (%) |  |  |  |  |  |  |
| Yes | 750(10.8)^a,b,c^ | 31(1.6)^d,e^ | 369(33.0)^f^ | 307(17.9) | 716.7 | <0.001 |
| No | 6165(89.2)^a,b,c^ | 1967(98.4)^d,e^ | 749(67.0)^f^ | 1405(82.1) |  |  |
| Cerebral hemorrhage, n (%) |  |  |  |  |  |  |
| Yes | 75(1.1)^b,c^ | 12(0.6)^d,e^ | 45(4.0) | 69(4.0) | 121.0 | <0.001 |
| No | 6840(98.9)^b,c^ | 1986(99.4)^d,e^ | 1073(96.0) | 1643(96.0) |  |  |
| Headache, n (%) |  |  |  |  |  |  |
| Yes | 322(4.7)^b,c^ | 97(4.9) | 75(6.7) | 113(6.6) | 16.7 | 0.001 |
| No | 6593(95.3)^b,c^ | 1901(95.1) | 1043(93.3) | 1599(93.4) |  |  |
| Heart disease , n (%) |  |  |  |  |  |  |
| Yes | 1036(15.0)^b^ | 314(15.7) | 205(18.3) | 300(17.5) | 12.4 | 0.005 |
| No | 5879(85.0)^b^ | 1684(84.3) | 913(81.7) | 1412(82.5) |  |  |
| MMSE score, median(IQR) | 28(26~29)^a,b,c^ | 23(21~25)^e^ | 23(21~25)^f^ | 14(10~16) | 7150.4 | <0.001 |
| ADL score, median(IQR) | 20(20~20)^a,b,c^ | 20(20~21)^e^ | 20(20~22)^f^ | 34(30~47) | 6547.1 | <0.001 |
| CDR score, median(IQR) | 0^a,b,c^ | 0.5^e^ | 0.5^f^ | 2(2~2) | 11703.6 | <0.001 |

Results are shown as n (%) for the chi-square tests and as the median(IQR) for Kruskal-Wallis test. TIA, transient ischemic attack; MMSE, mini-mental state examination; CDR, clinical dementia rating; ADL ,activities of daily living; IQR, inter quartile range. a, P < 0.05 normal vs. a-MCI; b, P < 0.05 Normal vs. na-MCI; c, P < 0.05 Normal vs. Dementia; d, P < 0.05 a-MCI vs. na-MCI; e, P < 0.05 a-MCI vs. Dementia; f, P < 0.05 na-MCI vs. Dementia. *Smoking was defined as having smoked at least 400 cigarettes. **Alcohol consumption was defined as drinking at least 0.1 drink per day for 1 year or more, with one drink equal to 10 g pure alcohol
